# Fabrication of Aligned Polyhydroxybutyrate Fibrous Scaffolds via a Touchspinning Apparatus

**DOI:** 10.1021/acsomega.4c11296

**Published:** 2025-05-25

**Authors:** Md Mazbah Uddin, Ummay Mowshome Jahan, Vijay Mohakar, Amit Talukder, Yahya Absalan, Brianna Blevins, Nataraja S. Yadavalli, Vladimir Reukov, Sergiy Minko, Suraj Sharma

**Affiliations:** † Department of Textiles, Merchandising, and Interiors, 1355 University of Georgia, 305 Sanford Dr., Athens, Georgia 30602, United States; ‡ Department of Textile Engineering, Chemistry, and Science, North Carolina State University, Raleigh, North Carolina 27606, United States; § Department of Chemistry, University of Georgia, 302 E Campus Rd suite 1299B, Athens, Georgia 30602, United States; ∥ CytoNest, Inc., 425 River Rd, Athens, Georgia 30602, United States

## Abstract

Poly­(3-hydroxybutyrate) (PHB) fibers ranging from nano- to microscale were successfully fabricated using a touchspinning apparatus. The optimization of key spinning parameters—including solution concentration (5–11% w/v), rotational speed (1300–2100 rpm), and feed rate (5–20 μL/min)—enabled the production of aligned fibrous scaffolds. Morphological analysis via field emission scanning electron microscopy (FE-SEM) revealed fiber diameters in the range of 0.831–1.273 μm, which were influenced by spinning conditions. Thermal stability was confirmed using thermogravimetric analysis (TGA), with an onset degradation temperature of ∼290 °C. Differential scanning calorimetry (DSC) showed a melting peak of ∼172 °C and a crystallinity increase from 37.9% in the pellet to 42.5% in fibers of PHB. The scaffolds were functionalized with collagen to enhance bioactivity, and fibroblast (NIH3T3) viability was assessed through alamarBlue and Live/Dead assays. Metabolic activity increased significantly over 5 days (*p* < 0.05), particularly in collagen-modified scaffolds, confirming excellent cell adhesion and proliferation. Immunofluorescent microscopy demonstrated cell elongation along the fiber axis, indicating scaffold-guided cellular orientation. The results establish the feasibility of touchspun PHB scaffolds for tissue engineering applications, offering a scalable alternative to the conventional electrospinning process.

## Graphical abstract

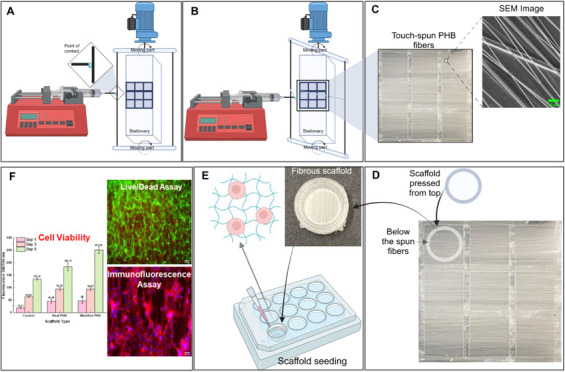

## 1 Introduction

Polymer nanofibers have emerged as a focal research area due to their excellent material properties, such as high surface area, tunable conductivity, and nanoscale dimensions. Their versatility has driven extensive research across diverse applications, including but not limited to tissue and bone regeneration,^1,2^ wound healing,^3,4^ drug delivery systems,^5^ biosensors,^6^ fuel cells,^7^ and advanced composite materials.^8,9^ Nanofibrous constructs are also known to possess enhanced transport^10^ and filtration properties,^11^ which can be leveraged in water and air purification systems, personal care products,^12^ and membranes.^13^

In tissue engineering and regenerative medicine, polymer nanofibers have been utilized for vascular grafts,^14^ artificial skin substitutes,^15^ and scaffolds for cartilage repair.^16^ Their ability to mimic the extracellular matrix (ECM) makes them particularly suitable for these bioengineering applications, where cell adhesion, proliferation, and differentiation are critical.^17^ Their high surface area, alignment, and porosity enhance interactions with biological systems, positioning them as ideal candidates for tissue engineering and regenerative medicine. For example, nanofibrous scaffolds loaded with growth factors have shown enhanced osteogenic differentiation for bone tissue engineering,^18,19^ while nanofibers functionalized with anti-inflammatory agents have been utilized to promote angiogenesis and tissue repair in wound healing models.^20^ Aligning nanofibers within scaffolds offers critical contact guidance that supports neurite outgrowth in primary cortical neurons and PC-12 cells, making these scaffolds particularly useful for spinal cord and peripheral nerve regeneration, where precise cell direction is essential for recovery.^21^ Furthermore, aligned nanofibers have shown promise in promoting myogenesis for skeletal muscle regeneration,^22^ demonstrating their broad utility in engineering various tissues. Studies have shown that aligned nanofibrous scaffolds can promote Schwann cell migration and axonal extension in nerve repair models.^23,24^ For instance, Niu et al. demonstrated that aligned polycaprolactone nanofibers significantly improved functional recovery in a rat sciatic nerve defect model.^25^

Researchers used several spinning techniques for developing polymeric nanofibrous scaffolds, including electrospinning and melt electrowriting. Electrospinning has emerged as the most widely adopted technology for producing nanofibrous scaffolds, primarily due to its ability to generate fibers with diameters as small as a few nanometers, closely resembling the fibrous structure of native ECM.^1,21^ Electrospinning employs high-voltage electric fields to stretch a polymer solution into fine fibers, collected into nonwoven mats with high surface area and porosity—fundamental properties supporting cell adhesion and proliferation, making them invaluable for tissue-engineered constructs. This technique has been broadly used in tissue engineering scaffold design as this versatile method can fabricate two-dimensional (2D) and three-dimensional (3D) fibrous structures.^26^

Despite its widespread adoption, electrospinning has its challenges. To promote cell adhesion, activity, and differentiation, nanofibers can be loaded with biomolecules such as bovine serum albumin (BSA) and growth factors such as heparin, which can be degraded due to the high voltages (typically 20–30 kV) used in the electrospinning system. Therefore, the bioactivity of the scaffolds becomes compromised, and their effectiveness in regenerative medicine becomes limited.^27^ Moreover, the resulting fibers produced from electrospinning are often randomly oriented fibers, which may not always be similar to the structure of the naturally produced extracellular matrix, affecting the mechanical properties and cell behavior on the scaffold.^28^ Achieving a uniform fiber diameter and distribution is another challenge of electrospinning, which may have an unfavorable effect on the reproducibility and performance of the scaffolds. These challenges have attracted interest in the development of alternative spinning techniques. Novel techniques like melt electrowriting can be an alternative to electrospinning, which offers precise control over fiber deposition and alignment.^29^ Spun fibers can be used in specialized applications such as muscle tissue engineering. However, this technique is limited by its slow fabrication rate and the potential for polymer degradation due to prolonged exposure to high temperatures.^30^ Therefore, there is a demand for scalable, safe, and efficient manufacturing processes that maintain the bioactivity of loaded molecules, which is a critical hurdle in the widespread application of nanostructured scaffolds in regenerative medicine.

The development of touchspinning techniques represents a significant innovation in this field. This technique is based on the principle of mechanical stretching of a polymer solution into nanofibers, which involves drawing nanofibers from the solutions using a rotating rod.^31,32^ This technique eliminates the need for high-voltage electric fields and allows for precise control of fiber diameter and orientation. Moreover, it provides flexibility in processing a wide range of polymer solutions, expanding its applicability across diverse biomedical and industrial fields. Also, unlike melt electrowriting or electrospinning, touchspinning preserves the structural and functional integrity of bioactive molecules, such as growth factors and enzymes, due to its gentler mechanical stretching mechanism.^33^ This feature makes it an excellent candidate for producing scaffolds loaded with bioactive molecules for advanced tissue engineering applications. Therefore, the touchspinning technique facilitates the production of nanofibers without the detrimental effects associated with electrospinning.^31^ Touchspinning has the potential to be scaled for industrial manufacturing, offering a safer and more efficient method for producing nanofibrous scaffolds.^33,34^

Among various polymers, polyhydroxybutyrate (PHB) stands out as a promising candidate for biomedical applications. Its unique combination of durability and biocompatibility makes it particularly well-suited for fabricating scaffolds used in tissue engineering.^12^ The electrospinning process is the widely adopted technique to spin electrospun PHB nanofibers for applications such as articular cartilage tissue regeneration.^13,14^ For instance, Movahedi and Karbasi developed a core–shell coaxial electrospun scaffold of PHB-starch/halloysite nanotubes containing ECM and chitosan, demonstrating suitability by enhanced mechanical properties and biocompatibility.^100^ Similarly, researchers have used PHB scaffolds in skin tissue engineering, where the nanofibrous structure supports fibroblast proliferation and collagen deposition. In a study by Ghavami et al., PHB scaffolds loaded with curcumin demonstrated improved anti-inflammatory properties and accelerated wound healing.^35^ Again, in bone tissue engineering, nanofibrous scaffolds can be designed to mimic the collagen fibers in the bone’s natural ECM, and these scaffolds have established their effectiveness in promoting the growth of new bone tissue by facilitating cell attachment, proliferation, and differentiation.^16^

Therefore, while electrospinning is the commonly preferred method for producing such scaffolds, the use of touchspinning for PHB fibers has yet to be reported. Hence, in this work, we employed the touchspinning technique to spin PHB fibers and optimized the spinning parameters to determine the most suitable conditions for producing touchspun (TS) fibers. Subsequently, TS fibers were assessed for their physical, thermal, and crystalline properties. Moreover, we conducted cell studies on NIH3T3 fibroblast cells using neat and collagen-modified scaffolds.

## 2 Materials and Methods

### 2.1 Materials

All of the materials were used as received. PHB, molecular weight of 550,000 g/mol (Good Fellow). Chloroform, ACS-certified reagent, 99.8% pure (Fischer Scientific). The following materials were used in the cell culture experiments: NIH3T3 (Cellbiolabs, INC), phosphate buffer solution (PBS, VWR Life Science), surface-treated standard tissue culture flask 75 cm^2^ (VWR), 12-well surface-treated standard tissue culture plates (VWR), DMEM (Dulbecco’s Modification of Eagle’s Medium) with 4.5 g/L glucose, l-glutamine, and sodium pyruvate (Corning Inc.), fetal bovine serum (FBS, Corning Inc.), antibiotic antimycotic solution (Sigma-Aldrich), 0.25% trypsin-EDTA solution (Sigma-Aldrich), alamarBlue cell viability assay (G-Biosciences), calcein AM (≥96.0% HPLC, Sigma-Aldrich) and ethidium homodimer-I (90% HPCE, Sigma-Aldrich) for live/dead, rhodamine phalloidin (Invitrogen) and Hoechst 33258 solution (1 mg/mL in H_2_O, Sigma-Aldrich) for cell staining, 4% paraformaldehyde (Alfa Aesar), and triton X-100 (Sigma-Aldrich). Collagen type-I, rat tail, 10 mg/mL (ibidi), for coating TS fibers.

### 2.2 Touchspun PHB Fiber Spinning

The spinning solution (% w/v) was prepared in an oil bath at 60–70 °C, with constant magnetic stirring at 500 rpm. To prevent solvent loss and pressure buildup, the solution was contained in a tightly sealed glass jar. Due to the polymer’s high molecular weight and crystallinity, elevated temperatures were necessary to fully dissolve it. A specialized touchspinning apparatus (Figure 1) was engineered to produce TS fibers. In the touchspinning process, droplets of the polymer solution are touched by high-speed rotating rods and mechanically drawn into thin strands. As the solvent evaporates, the wet strands crystallize and are subsequently collected onto fiber-collecting black frames placed on the spinning block of the instrument. Herein, TS fibers were spun using a 20G needle.

**Figure 1:**
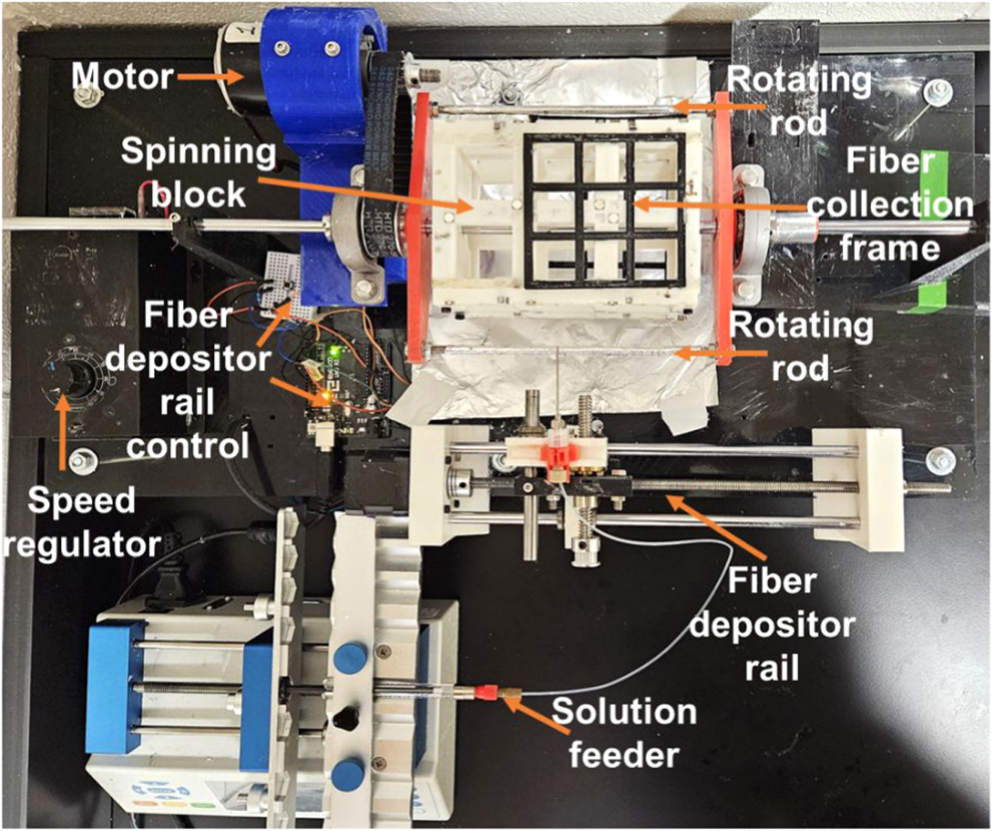
Touchspinning apparatus for the PHB scaffold fabrication.

The touchspinning device operates based on three key experimental parameters: polymer solution concentration (%), the rotational speed of the touching rod(s), and solution feed rate. We developed a spinning plan, as shown in Table 1, to find suitable conditions for fiber spinning with a set of nine experiments.

**Table 1:** Touchspinning of PHB Fibers

group A: effect of solution percentage on PHB TS fibers at a fixed speed and feed rate		
TS fibers spun with the different solutions at 1700 rpm and 5 μL/min	sample name	% w/v solution
	TS1	5
	TS2	7
	TS3	9
	TS4	11

group B: effect of solution feed rate on PHB TS fibers at fixed solution percentage and speed		
TS fibers spun with 7% w/v at 1700 rpm speed	sample name	feed rate (μL/min)
	TS2	5
	TS5	10
	TS6	15
	TS7	20

group C: effect of speed on PHB TS fibers at fixed solution percentage and feed rate		
TS fibers spun with 7% w/v at 10 μL/min	sample name	speed (RPM)
	TS8	1300
	TS5	1700
	TS9	2100

### 2.3 Collection of TS Fibers and Fabrication of the Scaffolds

3D-printed black frames were used to collect TS fibers for characterization and scaffold fabrication (Figures 1 and 2). As shown in Figure 1, the spinning block of the apparatus can hold four rectangular frames, namely, positions 1, 2, 3, and 4, in a clockwise manner (Videos S1 and S2). The fiber depositor rail of the instrument can move across the spinning block back and forth and deposit fibers on these black frames in a parallel manner. Herein, the fibers were deposited at a speed of 31 mm/min and for 2 h and 45 min. Three black frames from positions 1–3 were stacked in a unidirectional manner to create a denser fibrous mat (Figure 2a). Since fibers were breaking at position 4, they were excluded from preparing scaffolds. The final scaffolds were constructed using transparent ABS (acrylonitrile butadiene styrene) 3D-printed frames, specifically designed to fit into 12-well cell culture plates. The fiber mat formed in each square of the stacked black frames was then sandwiched between two clear ABS 3D-printed frames (bottom and top ABS frames) and secured using ABS glue (2 g/10 mL ABS in acetone) (Figure 2b,c). The bottom ABS frame had a trench where ABS glue was deposited, and afterward, stacked black frames were positioned over the bottom frame. The top scaffold frame had a ridge that fits into the trench of the bottom frame. Once all frames were aligned above each other appropriately (bottom frame > stacked frames > top frame), the top frame was pressed into the bottom frame with a glass vial of 27 mm in diameter (Figure 2d). Thereby, the fiber mat can be easily sandwiched between the two frames to make the final scaffolds once the acetone is evaporated (Figure 2e). The completed scaffolds had an internal diameter of 12.7 mm and a thickness of 12 μm, covering an area of 126.7 mm^2^. Additionally, collagen-coated scaffolds were prepared by treating neat PHB scaffolds with diluted collagen solutions of different concentrations, ranging from 2.0 to 0.05 mg/mL. The scaffolds were first rinsed twice with PBS, followed by treatment with diluted collagen solutions for 10 s, and then incubated at 37 °C for one h for drying. The collagen stock solution was diluted using 17.1 mM acetic acid solution, and before cell seeding, scaffolds were rinsed three times in PBS.^36–38^

**Figure 2:**
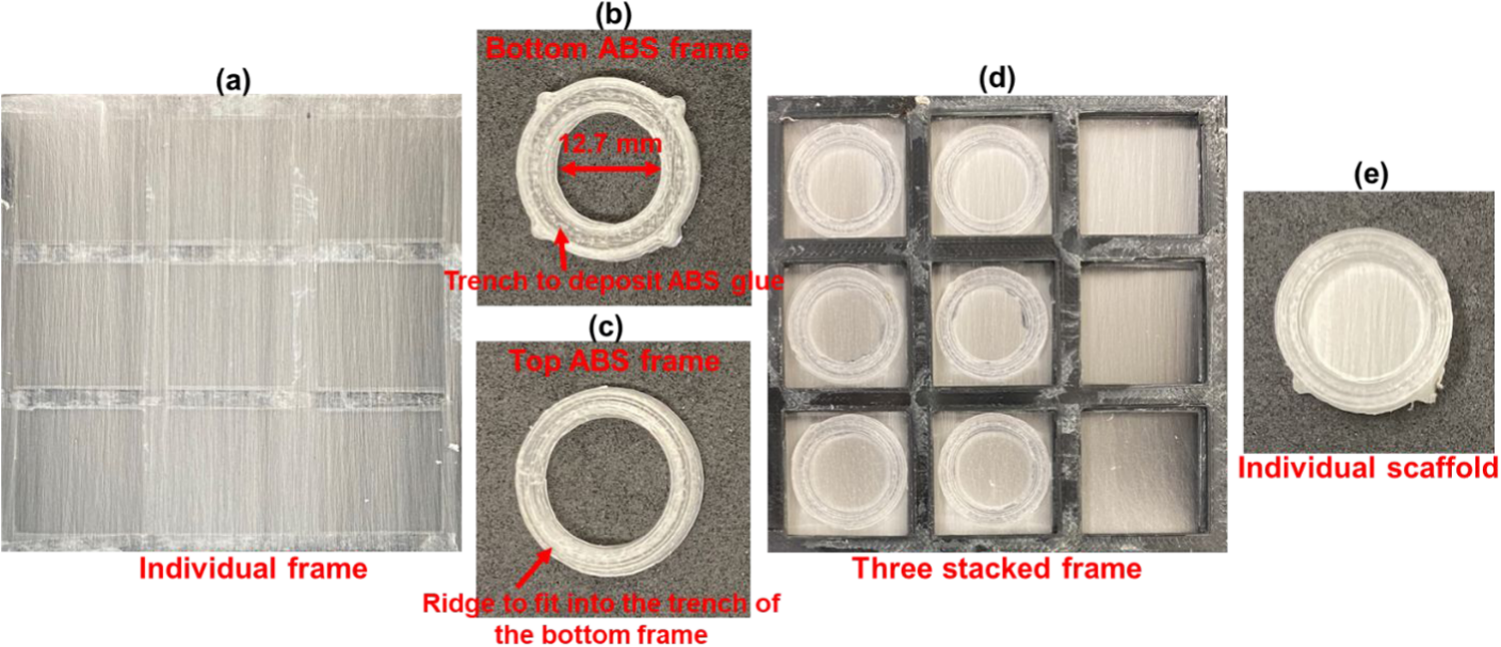
Fabrication of TS PHB fibrous scaffolds. (a) Collection of TS fibers, (b, c) transparent ABS printed bottom and top scaffold frames, (d) TS unidirectional fiber mat sandwiched between the ABS frames using ABS glue (2 g/10 mL ABS in acetone), and (e) final scaffold for cell culture studies.

### 2.4 Morphological, Thermal, and Crystallinity Characterization of the TS Fibers

The morphological characterization of the TS fibers was carried out in a field emission scanning electron microscope (FE-SEM), Thermo Fisher Scientific, at 5 kV operating voltage with 30 nm of Au/Pd coating using a Leica EM ACE600 Coater. Under the SEM electron beam, the TS PHB fibers were thermally degrading, and therefore, a lower voltage was chosen; even at that voltage, fibers were degrading, and the resolution was too low to differentiate between two fibers. Therefore, the same magnification and scale bar were hard to keep constant to achieve a better visual comparison. Thermogravimetric analysis (TGA) was carried out using PerkinElmer’s TGA 8000 to assess the fibers’ thermal stability. The operating temperature ranged from 30 to 850 °C at a rate of 20 °C per minute while using 20 mL/min of N_2_ gas. To measure the degree of crystallinity of the fibers, Differential Scanning Calorimetry (DSC) was performed using a PerkinElmer DSC 8000. The operating temperature was chosen between 30 and 200 °C at the rate of 10 °C min^–1^ under 20 mL/min^–1^ N_2_ gas. The average sample weight for DSC and TGA was 5.20 and 5.10 mg, respectively, and variation in sample weight was kept between ±10% for comparison. The curve was smoothened, and sigmoidal baseline correction was performed before thermal analysis. D2 Phaser benchtop X-ray diffractometer (XRD) from Bruker was used at 35 kV and 40 mA with a Cu Kα (λ = 1.54 A^o^) radiation source to take XRD spectra. The scanning rate and step size used for the XRD patterns were 0.2 s step^–1^ and 0.02°, respectively, at 2θ = 10–40°. Fourier-transform infrared spectroscopy (FTIR) studies were conducted on PerkinElmer’s Spectrum 3 at 32 scans and 4 cm^–1^ resolution using the ATR mode.

### 2.5 Cell Culture Studies on TS PHB Fibrous Scaffolds

#### 2.5.1 Cell Seeding

Fibroblast cells were cultured in DMEM containing 4.5 g/L glucose, supplemented with 10% FBS and 1% Penicillin-Streptomycin. The cells were grown in a 75 cm^2^ cell culture flask and incubated at 37 °C in a humidified atmosphere with 5% CO_2_. The culture medium was refreshed every 2 days. Once the cells reached 90% confluency, they were detached using 0.25% trypsin-EDTA and prepared for seeding into 12-well polystyrene (PS) 12-well plates. Before cell seeding, the scaffolds were UV-sterilized for 30 min and then washed with PBS three times. Cells were seeded at a density of 30,000 cells per well and monitored over 1, 3, and 5 days. Control wells, containing the same cell density but with no scaffolds, were also seeded.

#### 2.5.2 Cell Viability and Metabolic Assay

On days 1, 3, and 5, postseeding of cells on the control, neat PHB, and collagen-modified PHB scaffolds, cell viability and metabolic assays were conducted. At each time point, the scaffolds were transferred to new wells for cell viability and the metabolic assay.

Cell viability was assessed using the alamarBlue assay, with measurements taken after 2 h of incubation. Fluorescence was measured using a Varioskan LUX multimode microplate reader (Thermo Fischer Scientific) at an excitation wavelength of 540 nm and an emission wavelength of 590 nm. All experiments were conducted in triplicate. Live and dead cells were labeled using calcein AM (2 μM) and ethidium homodimer (4 μM) according to standard protocols. The controls and scaffolds were incubated with the stains for 30 min, and the cells were then imaged using an inverted fluorescence microscope (EVOS M5000) at 20× magnification. All experiments were performed in triplicate.

#### 2.5.3 Immunofluorescence Assay

For morphological analysis, cells were first fixed in 4% paraformaldehyde in PBS on the scheduled days. They were then stained for the cytoskeleton using 4 μM rhodamine phalloidin and for nuclei using 4 μM Hoechst, with incubation carried out for 60 min according to standard protocols. Immunofluorescence images were captured using an EVOS M5000 at 20× magnification, with multiple areas randomly selected from each sample. All experiments were performed in triplicate.

### 2.6 Statistical Analysis

Statistical analysis was conducted to validate the experimental results. One-way ANOVA (Analysis of Variance) was used to determine the significance of differences in metabolic activity between groups (Control, neat PHB, and collagen-modified PHB scaffolds) at each time point (days 1, 3, and 5). Tukey’s HSD (honestly significant difference) was performed for pairwise comparisons to identify specific group differences. Statistical significance was considered at a *p*-value <0.05. Results are presented as mean ± standard deviation. All statistical analyses were conducted using OriginPro 2024b (v10.1.5.132) software at a significance level (α) of 0.05, ensuring the reliability and validity of the data.

## 3 Results and Discussion

### 3.1 Characterizations of Morphological, Thermal, and Crystalline Properties of TS PHB Fibers

TS PHB fibers were spun using a combination of three key parameters: spinning solution concentration (% w/v), feed rate (μL/min), and spinning speed (rpm). These parameters were systematically varied to assess their influence on the fiber formation and properties. Figure 3 shows the SEM images of the TS fibers, and the insets illustrate the fiber diameter distributions derived from SEM of different TS fibers mentioned in Table 1. The data reveal a clear trend: the overall fiber diameter increases as the solution concentration and feed rate increase from 5 to 11% w/v and 5 to 20 μL/min, respectively, while other parameters remain constant. Specifically, fiber diameter increases from 0.940 to 1.176 μm with increasing solution concentration at a constant speed (1700 rpm) and feed rate (5 μL/min) and from 0.874 to 1.273 μm with an increased feed rate at a constant speed (1700 rpm) and solution concentration (7% w/v) (Figure 4a,b). Conversely, a decrease in average fiber diameter from 0.989 to 0.831 μm is observed with higher spinning speeds from 1300 to 2100 rpm (Figure 4c) at a constant solution concentration (7% w/v) and feed rate (10 μL/min).^39^ At higher concentrations of 9 and 11% w/v PHB, the solution solidifies rapidly at the needle tip due to the lower chloroform to PHB ratio. Higher concentrations lead to a denser solution, which solidifies more quickly than a less concentrated solution when exposed to air. This rapid solidification causes frequent needle blockages and forms unwanted flimsy residues on the collected fibers during the touchspinning process.^40,41^ According to the statistical analysis (Tables S1–S3 and Figure 4), no significant difference in fiber diameter was observed between TS1 and TS2 or TS3 and TS4 (Figure 4a) in Group A, TS2 and TS5 (Figure 4b) in Group B, and TS5 and TS9 (Figure 4c) in Group C from Table 1 at a 0.05 confidence level. All other comparisons within each group showed statistically significant differences at the same confidence level.

**Figure 3:**
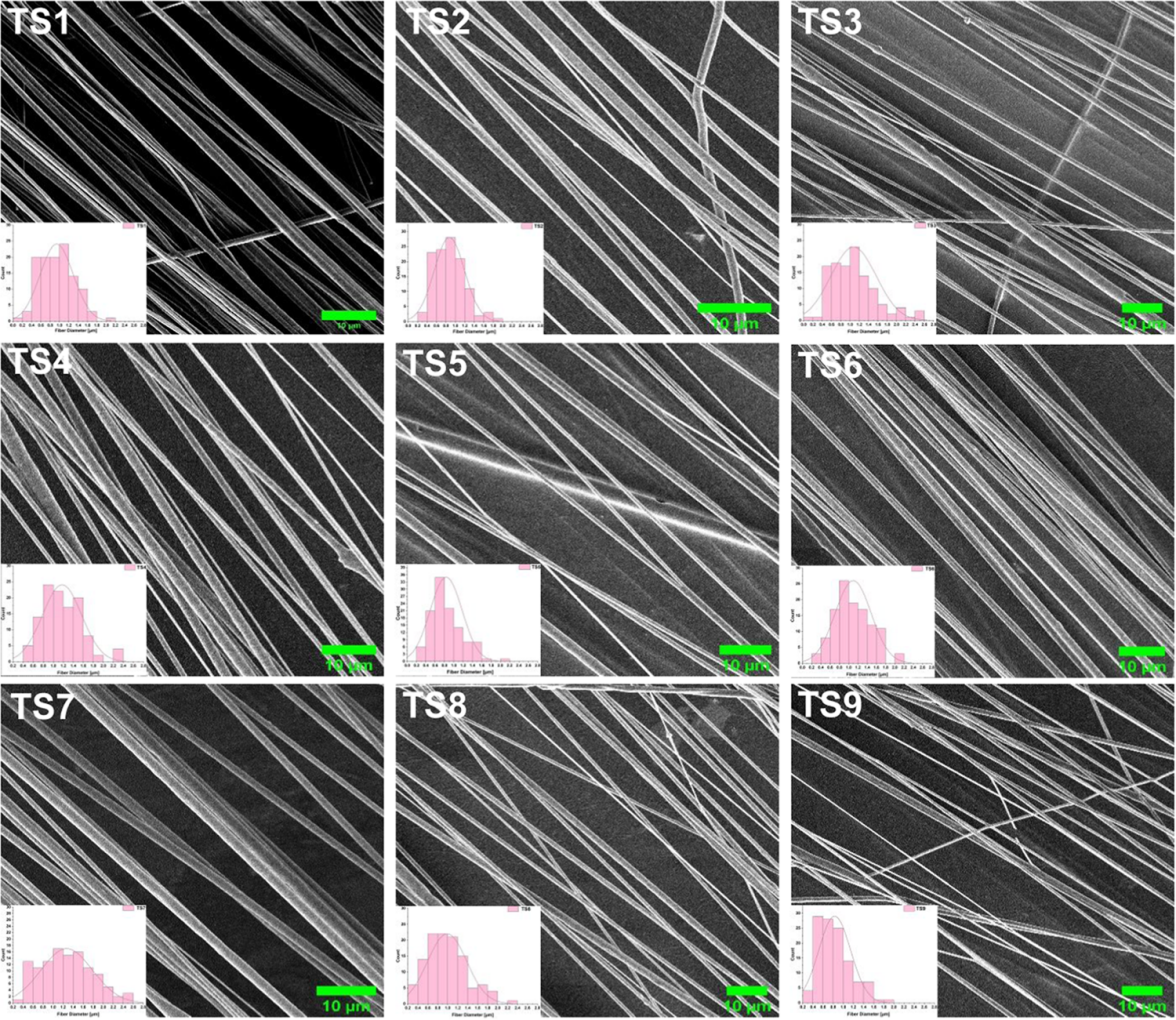
SEM images of the TS PHB fibers. TS1–TS4 fibers were spun using 5, 7, 9, and 11% w/v PHB solutions at 1700 rpm and a feed rate of 5 μL/min. TS5–TS7 fibers were spun with a 7% w/v PHB solution at 1700 rpm, with feed rates of 10, 15, and 20 μL/min, respectively. TS8 and TS9 fibers were spun with a 7% w/v PHB solution at 10 μL/min with 1300 and 2100 rpm, respectively (Scale bar: 10 μm).

**Figure 4:**
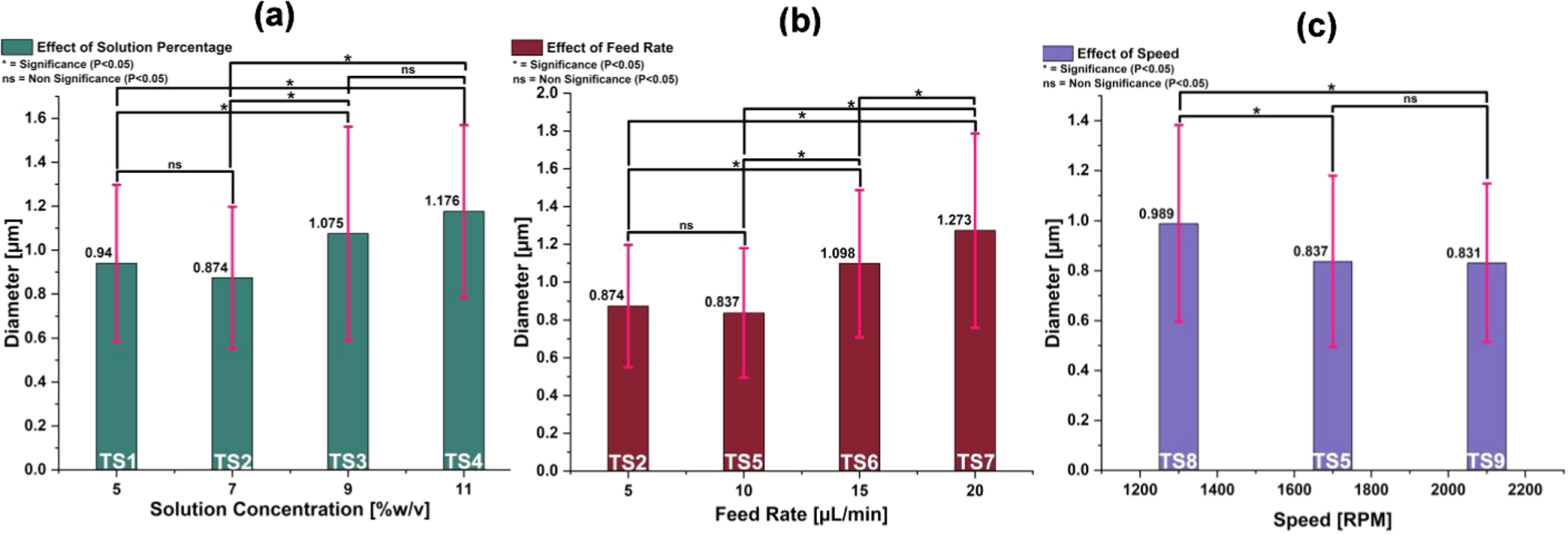
Summary of SEM analysis of the TS PHB fibers. Effect of (a) solution percentage on fiber diameter at 1700 rpm and 5 μL/min, (b) feed rate at 7% w/v and 1700 rpm, and (c) speed at 7% w/v and 10 μL/min.

For scaffold fabrication, the speed was chosen to be maintained at 1700 rpm to withhold the mechanical integrity of the touchspinning apparatus. However, with more robust engineering, this speed limitation could be addressed, potentially allowing for further reduction of fiber size if desired. Consequently, to address the aforementioned issues, spinning with a 7% w/v PHB solution at a feed rate of 10 μL/min and a rod speed of 1700 rpm was found to be the optimal condition for producing TS fibers and fabricating scaffolds (i.e., TS5). Furthermore, while the commonly used electrospinning technique depends on several key parameters such as solution concentration, spinning speed, and feed rate, it is also influenced by additional process variables. These include the dielectric properties of the solution, stable jet formation, the distance between the needle and collector, and the applied voltage.^42^ In contrast, our touchspinning apparatus is significantly straightforward and confined to only three variables, highlighting its advantages over the electrospinning process. This enabled us to achieve greater control over fiber collection, alignment, and arrangement without any sophisticated instrumentation compared to electrospinning.

The thermal and crystalline properties of the fibers were analyzed using TGA and DSC analysis at 20 and 10 °C per minute, respectively, under the N_2_ atmosphere. TGA analysis (Figure 5a,b and Table 2) of the TS fibers reveals no significant differences between the PHB pellet and the different TS fibers mentioned in Table 1. For all samples, the onset temperature and inflection point (IP) remain around 290.3 and 307.8 °C, respectively, with similar amounts of mass loss of about 98.7%, which is similar to what was reported in the literature for electrospun PHB fibers between 200 and 350 °C.^42,43^ Furthermore, all samples exhibited a degradation peak around 405 °C, which can be attributed to the presence of additional organic additives in as-received pellets, as reported by Follain et al., and this peak may not appear for all grades of PHB mentioned in the literature.^43,44^

**Figure 5:**
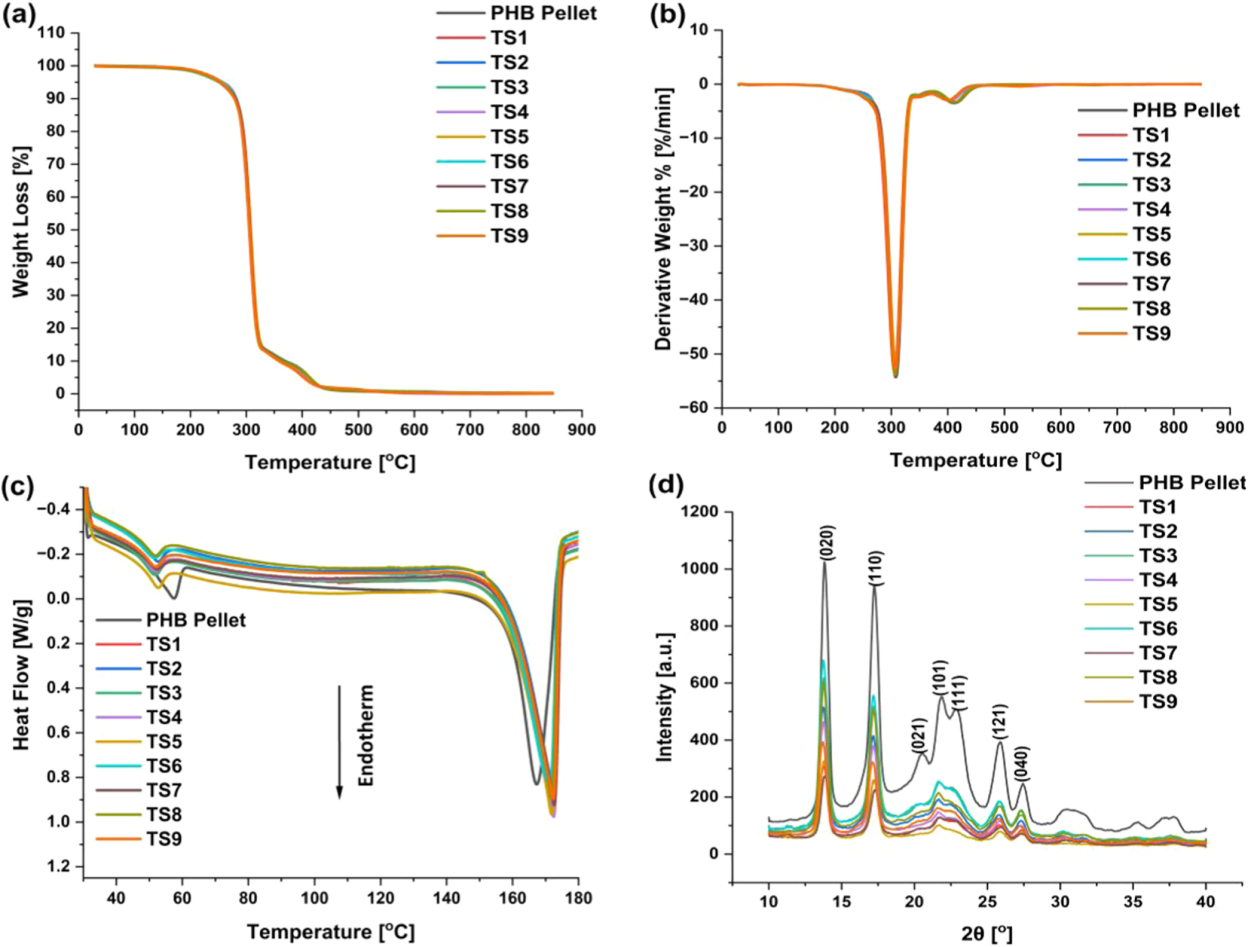
Thermal and crystallinity measurements of TS PHB fibers. (a) % Weight loss and (b) derivative curves from TGA. (c) DSC and (d) XRD profiles of the TS PHB fibers.

**Table 2:** Summary of the TGA and DSC Analyses of TS PHB Fibers

	TGA analysis				DSC analysis			
Sl#	onset (°C)	% mass at onset	inflection point (IP) (°C)	% mass loss at IP	onset (°C)	melting peak (°C)	Δ*H* (J/g)	% *X*_c_
PHB pellet	290.83	99.77	308.86	98.53	159.06	167.5	55.31	37.88
TS1	288.01	99.62	306.47	98.56	161.35	171.7	60.93	41.73
TS2	289.55	99.63	308.23	98.32	161.44	172.38	63.32	43.37
TS3	289.18	99.76	306.98	98.31	161.3	171.81	62.88	43.07
TS4	290.60	99.64	308.06	98.98	163.35	172.49	63.05	43.18
TS5	290.2	99.63	308.73	98.70	160.86	171.95	63.48	43.48
TS6	290.91	99.29	307.94	98.60	158.19	171.14	63.54	43.52
TS7	291.85	99.66	307.94	98.98	162.7	172.63	61.93	42.42
TS8	290.82	99.75	306.89	98.85	161.96	171.73	62.51	42.82
TS9	290.73	99.66	307.44	99.15	159.55	172.34	63.17	43.26

DSC analysis (Figure 5c and Table 2) reveals that TS fibers exhibit a higher crystallinity of 42.50% compared with 37.90% for PHB pellets. The degree of crystallinity of different PHB samples was calculated using eq 1, where Δ*H*_s_ is the melting enthalpy of fusion from DSC thermograms of the sample and ΔH_100_ is the enthalpy of fusion for 100% crystalline PHB, which is 146 J/g for PHB.^45^

1 $${\%\;X}_{c}=\frac{{\Delta H}_{s}}{{\Delta H}_{100}}\times 100$$

Additionally, the melting point of TS fibers (172 °C) is slightly higher than that of the PHB pellet (167.5 °C). The onset of melting (161 °C) is consistent across all samples, with an additional peak observed at 53 °C, which can be attributed to the presence of organic additives.^44,45^ The increased crystallinity in the TS fibers can be attributed to the mechanical stretching applied during processing.^33,46^ The crystallinity of electrospun PHB fibers is typically reported to range between 38.3 and 63.2% when measured using DSC. Neat PHB pellets generally exhibit higher crystallinity, a trend that aligns with our DSC analysis.^47–49^ Thermal properties observed in TGA and DSC analysis can vary depending on the specific PHB grade and molecular weight.^50^ Additionally, these analyses often require baseline correction, curve smoothing, and different data interpretation methods to extrapolate different thermal events, all of which can significantly impact the reported value of the thermal properties.^51,52^ Therefore, it is essential to specify the curve processing parameters used in the final analysis to ensure accurate comparisons. Nonetheless, our apparatus can produce fibers with morphology, thermal, and crystalline properties comparable to those of electrospun PHB fibers. The ability to produce PHB fibers without the need for melting or high-voltage applications enables the seamless incorporation of thermally and electrically sensitive biomolecules into these fibers.

The XRD spectra of TS fibers and the PHB pellet are presented in Figure 5d. XRD spectra of fibers exhibited similar peak positions and intensities, indicating that both conformations share a similar crystalline phase. Both α and β-form crystalline forms of PHB are present in all samples. In the PHB pellet, a relatively intense β-form was observed, while in TS fibers, only a very mild shoulder peak from the β-form appears.^53^ XRD peaks corresponding to orthorhombic crystal planes of (020), (110), (021), (101), (111), and (121) at 2θ values of 13.84, 17.24, 20.5, 21.86, 22.92, 25.86, and 27.49°, respectively, were found to be similar for all samples.^54,55^ In FTIR, the asymmetric C–H stretching of the methyl (−CH_3_) group was observed at 2976 cm^–1^, while that of the methylene (−CH_2_−) group appeared at 2935 cm^–1^. Additionally, symmetric C–H vibrations from both −CH_3_ and −CH_2_ groups were detected around 2876 and 2853 cm^–1^, respectively. The characteristic stretching vibration of carbonyl (−C=O) was observed at 1720 cm^–1^, and the C–O stretching vibrations were observed at 1054 and 1277 cm^–1^. These bands confirm the presence of PHB polymer-based materials (Figures 6a and S1a).^56^ Additionally, scaffolds were dip-coated with collagen for better cell adhesion and support.^57^ When modified with collagen, in Figure 6, the characteristic C=O stretching vibration from amide I, C–N stretching and N–H bending of amide II, and N–H bending and C–N stretching were observed for type-1 collagen at 1630, 1544, and 1233 cm^–1^, respectively.^58^ The N–H stretching vibrations from Amid A at 3297 cm^–1^ and Amide B at 2938 cm^–1^ were observed. The appearance of such bands confirms the presence of collagen coatings on PHB TS fibers (Figures 6b­(i–iii) and S1b,c).^59,60^

**Figure 6:**
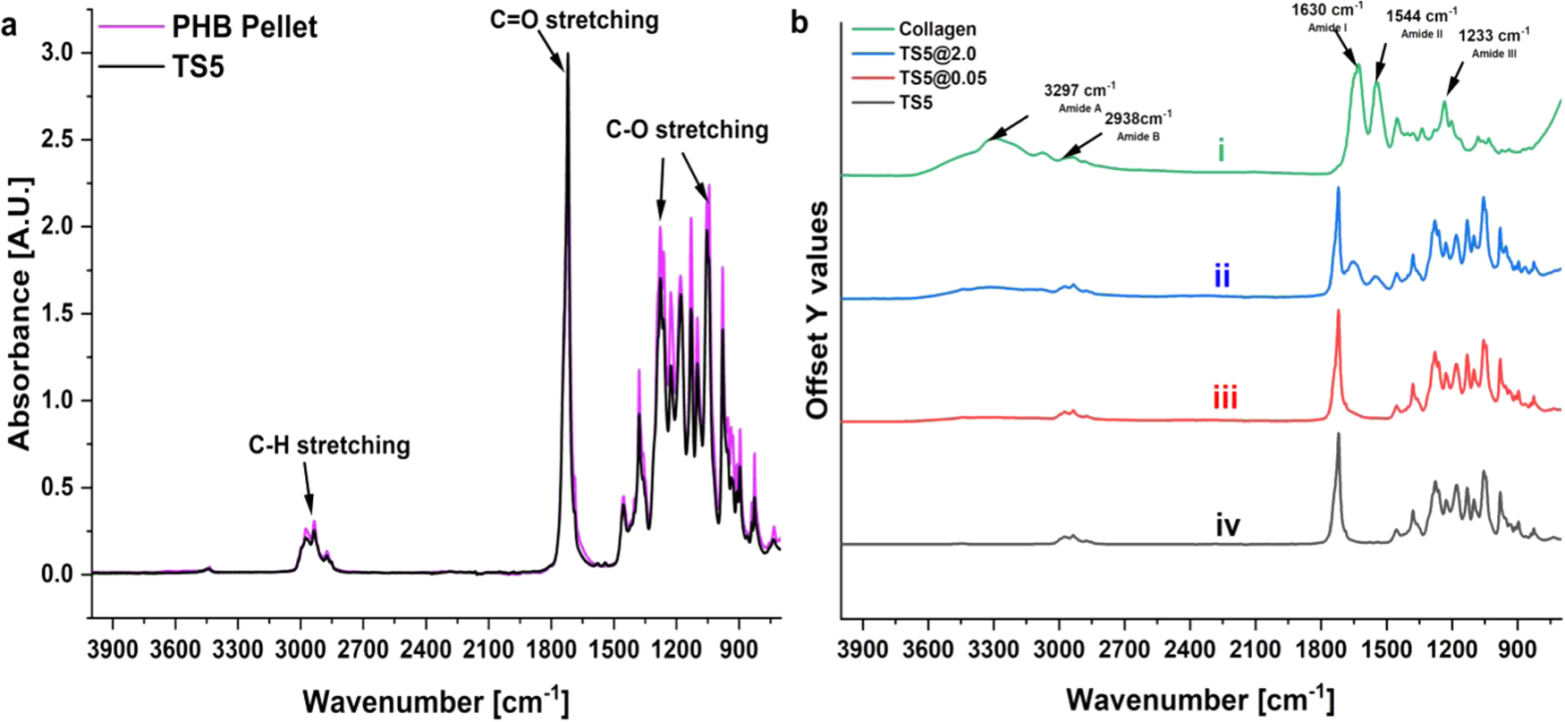
FTIR spectra of PHB pellet, TS5, and collagen-treated TS5 fibers of PHB. PHB fibers. (a) PHB pellet and (a, b­(iv)) TS5 fibers. (b­(i)) Neat collagen, (b­(ii)) TS5 treated with 2.0 mg/mL, and (b­(iii)) 0.05 mg/mL collagen solutions.

### 3.2 Cell Adhesion, Viability, and Proliferation Studies on PHB TS Scaffolds

The scaffolds were treated with collagen to improve cell attachment and proliferation.^61,62^ They were immersed in collagen solutions of various concentrations (mg/mL) for 10 s and then incubated at 37 °C for one h to evaluate the presence and integration of collagen on the fibers. Optical microscopy images (Figure S2) confirmed that collagen was successfully deposited on the scaffolds. A visible film layer formed on the fibers at higher collagen concentrations, while the lowest concentration maintained the fibrous structure without forming a film. Higher collagen concentrations disrupted fiber exposure and potentially hindered cell-scaffold interactions, while the optimized 0.05 mg/mL concentration maintained the scaffold’s structural integrity and biocompatibility.

Fluorescent microscopy and Live/Dead assays (Figure 7) provided qualitative evidence of cell adhesion and viability on both neat PHB and collagen-coated PHB scaffolds. On days 1 and 3, cells adhered to both scaffolds with no observable dead cells, confirming the absence of cytotoxicity at these early time points. By day 5, cell coverage was increased on all scaffolds, indicating that the scaffolds provided a supportive environment for cell growth. The collagen-coated scaffold showed better cell adhesion and spreading, likely due to its biomimetic extracellular matrix-like properties, which provide a physiologically favorable environment for cell growth.^63,64^

**Figure 7:**
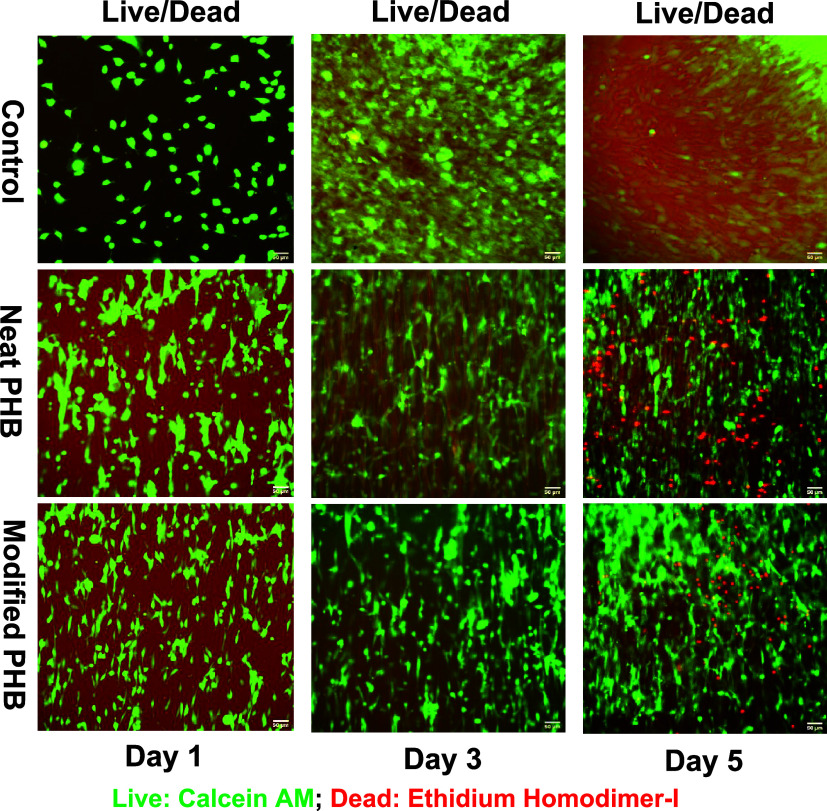
Viability of fibroblast cells on the control and PHB scaffolds. In merged images, live cells (green, calcein AM) and dead cells (red, ethidium homodimer-I) are shown over days 1, 3, and 5 using the inverted fluorescence microscope (EVOS M5000) at 20× magnification (Scale bar: 50 μm).

The alamarBlue assay (Figure 8) quantified metabolic activity, directly reflecting cell viability and proliferation on the scaffolds.^65^ Both neat PHB and collagen-coated scaffolds showed significantly higher metabolic activity than the control group (2D well plates) across all time points, as confirmed by one-way ANOVA and Tukey’s HSD test. This quantitative evidence supports the observations from Figure 7, which show increasing cell numbers on the scaffolds. By day 5, the metabolic activity on the collagen-coated scaffold was significantly higher than both the neat PHB scaffold and the control. This finding corroborates the lower number of dead cells and higher cell density observed qualitatively in Figure 7, emphasizing the scaffold’s biocompatibility. Additionally, the steady increase in metabolic activity from the alamarBlue assay, particularly on the 3D scaffolds, indirectly suggests that cells are proliferating and likely migrating across scaffold layers due to enhanced interactions with the 3D architecture.^66^

**Figure 8:**
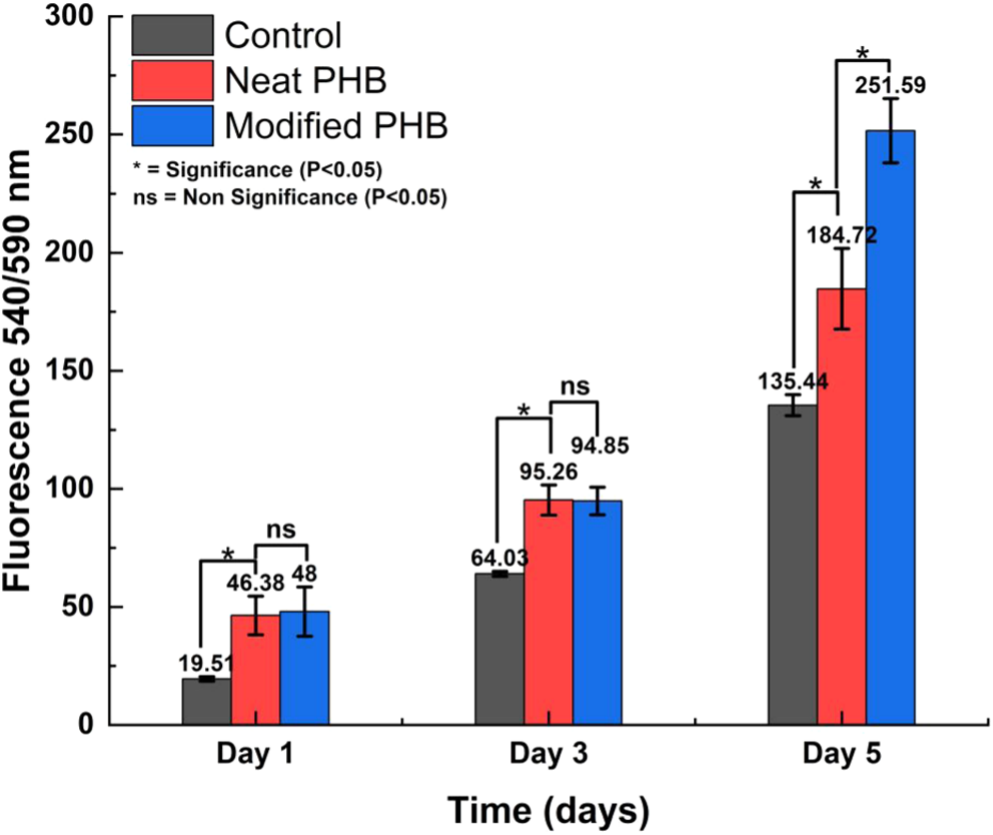
Metabolic activity of the fibroblast cells on control and PHB scaffolds at days 1, 3, and 5 using a Varioskan LUX multimode microplate reader (Thermo Fischer Scientific) at an excitation wavelength of 540 nm and an emission wavelength of 590 nm. The error bars represent the standard deviation.

Immunofluorescence microscopy (Figure 9) revealed morphological differences in fibroblast cells cultured on the scaffolds. Cells on both neat and collagen-coated scaffolds exhibited an elongated, spindle-shaped morphology, aligning along the fiber direction. This indicates that the nanofibrous architecture provides physical cues that guide cellular behavior.^22,67^ On the other hand, cells cultured on the 2D control plate exhibited random alignment and significantly less elongation. The spindle-shaped cells on the scaffolds suggest that the 3D architecture effectively mimics the in vivo extracellular matrix, further supporting cell attachment and directional behavior essential for tissue engineering applications such as muscle, tendon, and nerve regeneration.^68,69^

**Figure 9:**
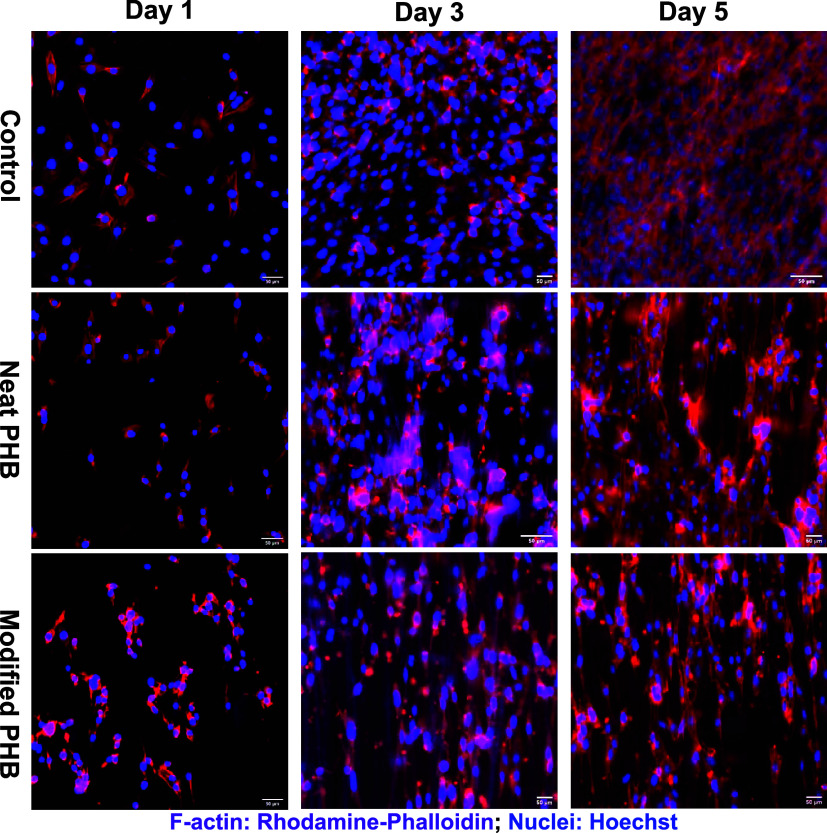
Morphology of fibroblast cells on the control and PHB scaffolds. F-actin (purple, Rhodamine phalloidin) and nuclei (blue, Hoechst) visualize cell morphology on control, neat, and collagen-modified scaffolds at days 1, 3, and 5 using the inverted fluorescence microscope (EVOS M5000) at 20× magnification (Scale bar: 50 μm).

The structural properties of scaffolds play a crucial role in directing cell behavior. The directional elongation of cells cultured on nanofibrous scaffolds indicates that the aligned structure significantly influences cellular alignment and organization.^70,71^ This phenomenon is particularly important for tissue engineering applications where cellular orientation is essential, such as in skeletal muscle, tendons, and nerve regeneration. This study highlights the flexibility of the touchspinning technique over traditional methods like electrospinning for fabricating PHB 3D fibrous scaffolds in specific alignment and arrangement.^72^ However, some limitations remain; for instance, our study was restricted to unidirectional fiber alignment. Exploring different 3D fiber arrangements is necessary to optimize the scaffold’s overall performance for specific tissue engineering applications. Additionally, while the collagen coating enhances biocompatibility, it may impact fiber stability and mechanical properties over time. Long-term studies are needed to assess the mechanical behavior of these scaffolds. Future research should also focus on integrating bioactive molecules, such as growth factors or drugs, to create controlled bioactive environments tailored to specific tissue types. In-vitro studies with various cell types, along with in vivo experiments, will provide deeper insights into the scaffold’s versatility and effectiveness for regenerative medicine applications.

## 4 Conclusions

This study successfully demonstrated the fabrication and optimization of aligned PHB fibrous scaffolds using a touchspinning apparatus, eliminating the need for high-voltage electrospinning. We achieved aligned fibrous scaffolds with tunable properties by optimizing key parameters, including solution concentration (5–11% w/v), feed rate (5–20 μL/min), and rod speed (1300–2100 rpm). The resulting touchspun PHB fibers displayed favorable morphological (average fiber diameter between 0.831 and 1.273 μm), thermal (∼290 °C onset degradation), and crystalline properties (∼42.5%) similar to electrospun PHB fibers and were able to maintain their structural integrity and thermal stability during cell culture studies. The biocompatibility of the scaffolds was confirmed through a cell viability assay and metabolic activity measurements. Functionalization of the scaffolds with collagen significantly enhanced their biocompatibility, which is shown by increased adhesion, proliferation, and migration of the fibroblast cells. Both scaffolds exhibited significantly higher metabolic activity than the control at all time points, and statistical analysis confirmed these differences at a 0.05 confidence level. The collagen-coated scaffold showed increased metabolic activity by day 5, significantly outperforming the neat PHB scaffold. The biomimetic properties of the collagen coating are responsible for mimicking the extracellular matrix and providing a conducive environment for cell adhesion, proliferation, and migration. Immunofluorescence microscopy demonstrated morphological variations in fibroblast cells cultured on the scaffolds. On both the unmodified and collagen-coated scaffolds, the cells displayed an elongated, spindle-like shape, aligning with the fiber orientation. This suggests that the nanofibrous structure offers physical cues that influence the cellular behavior. These findings proved that a modified PHB scaffold can support long-term cellular activity and has great potential in tissue engineering applications.

## Supplementary Material







## Data Availability

All data supporting this study’s findings are included in the article.

## Notes

Statistical analyses of fiber size and cell studies; optical microscopic images and additional FTIR spectra related to the modification of the PHB materials (PDF)
Description of the touchspinning apparatus (Video S1) (MP4)
Spinning block where fibers are positioned during the spinning process (Video S2) (MP4)
