# Supplementary material for: Fabrication of Aligned Polyhydroxybutyrate Fibrous Scaffolds via a Touchspinning Apparatus
Source: ACS Omega. 2025 May 25;10(22):22735–46. doi: 10.1021/acsomega.4c11296 (PMC12163700; doi:10.1021/acsomega.4c11296)
Supplement: Supplementary file 1 [file ao4c11296_si_001.pdf]

# Supporting Information

## Fabrication of Aligned Polyhydroxybutyrate Fibrous Scaffolds via Touch-Spinning Apparatus

*[1]Md Mazbah Uddin, [2]Ummay Mowshome Jahan, [1]Vijay Mohakar, [1]Amit Talukder, [3]Yahya Absalan, [3]Brianna Blevins, [4]Nataraja S. Yadavalli, [1]Vladimir Reukov, [1,3]Sergiy Minko, [1,*]Suraj Sharma*

*[1]Department of Textiles, Merchandising, and Interiors, University of Georgia, 305 Sanford Dr., Athens, GA 30602, USA*
*[2]Department of Textile Engineering, Chemistry, and Science, North Carolina State University, Raleigh, North Carolina 27606, United States*
*[3]Department of Chemistry, University of Georgia, 302 E Campus Rd suite 1299B, Athens, GA 30602, United States*
*[4] CytoNest, Inc., 425 River Rd, Athens, GA 30602*

*Corresponding author: ssharma@uga.edu (Suraj Sharma)

**Table S1.** Effect of solution percentage on PHB TS fibers at a fixed speed and feed rate.

| Tukey Test | Mean Diff | SEM | q Value | Prob | Alpha | Sig | LCL | UCL |
|---|---|---|---|---|---|---|---|---|
| TS2 - TS1 | -0.06587 | 0.05184 | 1.79709 | 0.58207 | 0.05 | 0 | -0.1995 | 0.06779 |
| TS3 - TS1 | 0.13467 | 0.05184 | 3.67376 | 0.04752 | 0.05 | 1 | 0.001 | 0.26833 |
| TS3 - TS2 | 0.20054 | 0.05184 | 5.47085 | 7.21E-04 | 0.05 | 1 | 0.06687 | 0.33421 |
| TS4 - TS1 | 0.23545 | 0.05184 | 6.42314 | <0.0001 | 0.05 | 1 | 0.10178 | 0.36911 |
| TS4 - TS2 | 0.30132 | 0.05184 | 8.22022 | <0.0001 | 0.05 | 1 | 0.16766 | 0.43499 |
| TS4 - TS3 | 0.10078 | 0.05184 | 2.74938 | 0.21116 | 0.05 | 0 | -0.0329 | 0.23445 |

*Sig 1 means the difference between the means is significant at the significant level of 0.05*
*Sig 0 means the difference between the means is not significant at the significant level of 0.05*

**Table S2.** Effect of solution feed rate on PHB TS fibers at fixed solution% and speed.

| Tukey Test | Mean Diff | SEM | q Value | Prob | Alpha | Sig | LCL | UCL |
|---|---|---|---|---|---|---|---|---|
| TS5 - TS2 | -0.03757 | 0.0524 | 1.01398 | 0.89033 | 0.05 | 0 | -0.1727 | 0.09753 |
| TS6 - TS2 | 0.22368 | 0.0524 | 6.03731 | 1.40E-04 | 0.05 | 1 | 0.08858 | 0.35878 |
| TS6 - TS5 | 0.26124 | 0.0524 | 7.05129 | <0.0001 | 0.05 | 1 | 0.12615 | 0.39634 |
| TS7 - TS2 | 0.39825 | 0.0524 | 10.7493 | <0.0001 | 0.05 | 1 | 0.26315 | 0.53335 |
| TS7 - TS5 | 0.43582 | 0.0524 | 11.7633 | <0.0001 | 0.05 | 1 | 0.30072 | 0.57092 |
| TS7 - TS6 | 0.17458 | 0.0524 | 4.71203 | 0.00514 | 0.05 | 1 | 0.03948 | 0.30968 |

*Sig 1 means the difference between the means is significant at the significant level of 0.05*
*Sig 0 means the difference between the means is not significant at the significant level of 0.05*

**Table S3.** Effect of speed on PHB TS fibers at fixed solution% and feed rate.

| Tukey Test | Mean Diff | SEM | q Value | Prob | Alpha | Sig | LCL | UCL |
|---|---|---|---|---|---|---|---|---|
| TS9 - TS8 | -0.152 | 0.04626 | 4.64692 | 0.00321 | 0.05 | 1 | -0.26088 | -0.04311 |
| TS5 - TS8 | -0.15805 | 0.04626 | 4.83183 | 0.00205 | 0.05 | 1 | -0.26693 | -0.04916 |
| TS5 - TS9 | -0.00605 | 0.04626 | 0.18492 | 0.99062 | 0.05 | 0 | -0.11493 | 0.10284 |

*Sig 1 means the difference between the means is significant at the significant level of 0.05*
*Sig 0 means the difference between the means is not significant at the significant level of 0.05*

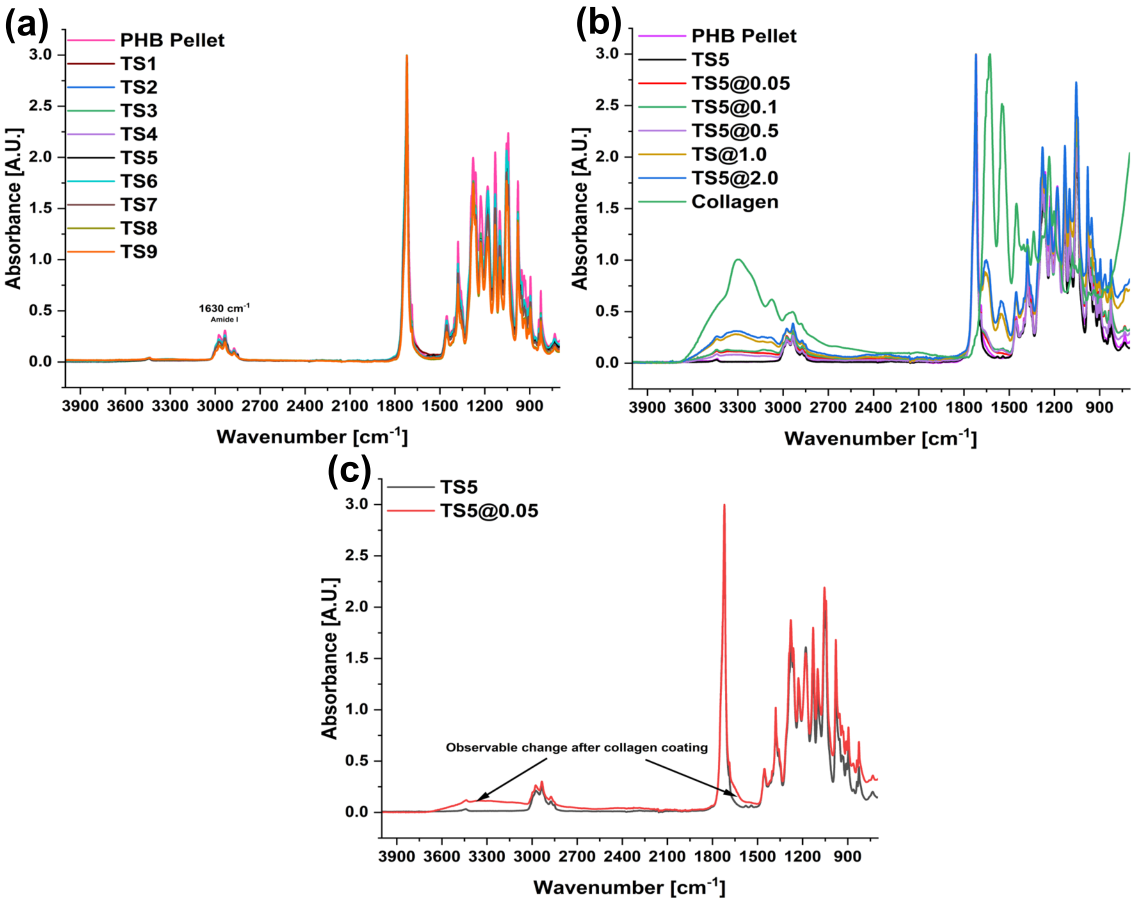


**Figure S1.** FTIR spectra of different PHB materials. (a) FTIR spectra of PHB pellet and TS fibers, (b) FTIR spectra of collagen and collagen modified TS5 fibers, and (c) FTIR Spectra of TS5 fibers with final collagen modification used for cell culture studies.

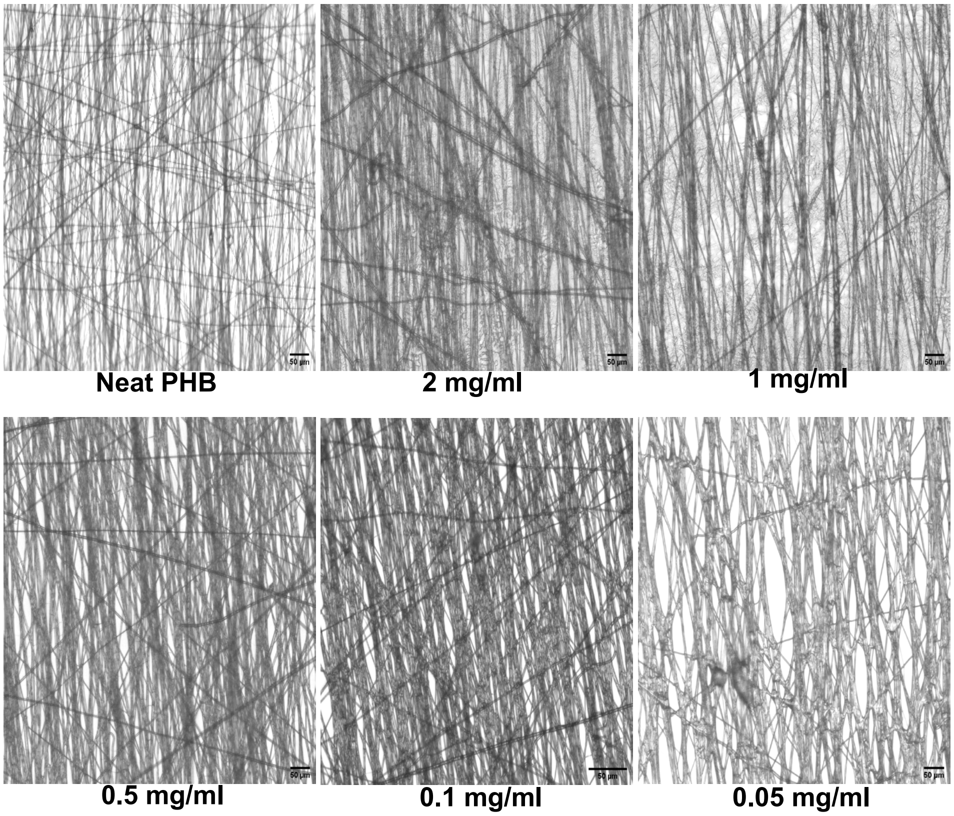


**Figure S2.** Optical images of PHB TS5 fibers with collagen modification.

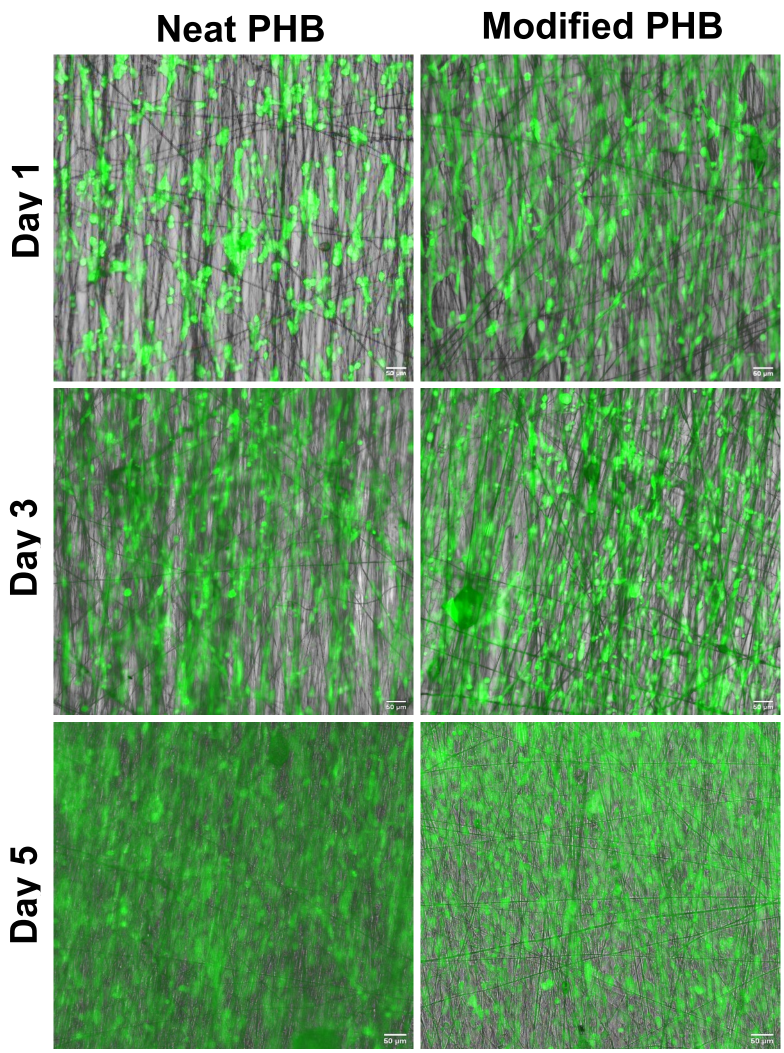


**Figure S3.** Fibroblast cells on PHB TS5 fibers.

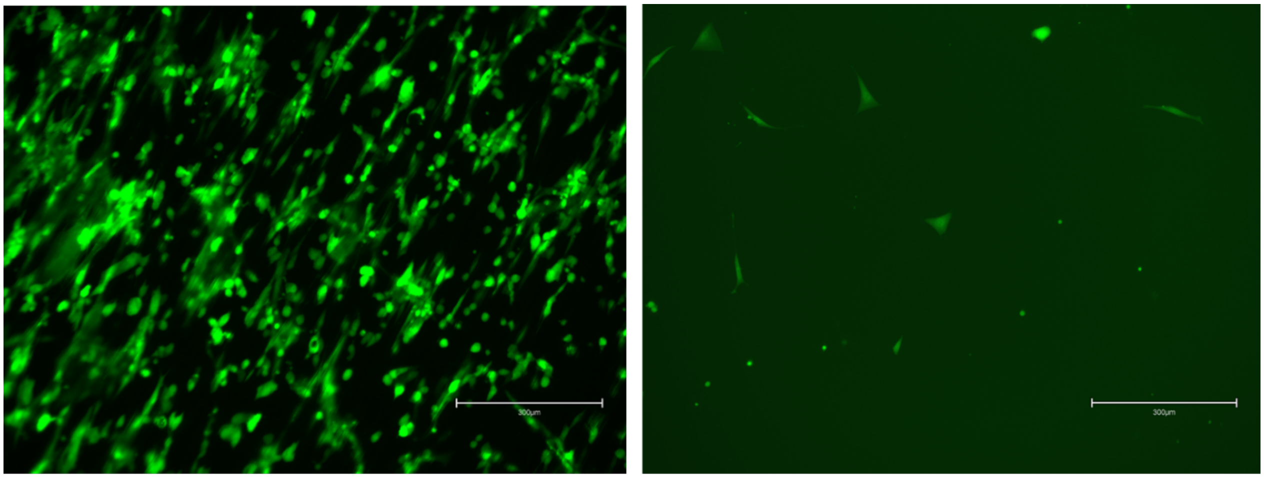

Cells on denser PHB scaffold

Fewer cells at the bottom of the well plate

**Figure S4.** Fibroblast cells on PHB TS5 fibers and control after day 1, cell-seeded at 30,000 cells per well.

**Table S4.** Statistical analysis of alamarBlue assay on day 1.

| Tukey Test | Mean Diff | SEM | q Value | Prob | Alpha | Sig | LCL | UCL |
|---|---|---|---|---|---|---|---|---|
| Neat PHB - Control | 27.22303 | 6.27827 | 6.13213 | 0.0116 | 0.05 | 1 | 7.95911 | 46.48695 |
| Modified PHB - Control | 28.8497 | 6.27827 | 6.49855 | 0.00884 | 0.05 | 1 | 9.58577 | 48.11362 |
| Modified PHB - Neat PHB | 1.62667 | 6.27827 | 0.36642 | 0.96389 | 0.05 | 0 | -17.6373 | 20.89059 |

*Sig 1 means the difference between the means is significant at the significant level of 0.05*
*Sig 0 means the difference between the means is not significant at the significant level of 0.05*

**Table S5.** Statistical analysis of alamarBlue assay on day 3.

| Tukey Test | Mean Diff | SEM | q Value | Prob | Alpha | Sig | LCL | UCL |
|---|---|---|---|---|---|---|---|---|
| Neat PHB - Control | 32.04514 | 5.15029 | 8.79925 | 0.00365 | 0.05 | 1 | 15.28626 | 48.80401 |
| a | 31.63569 | 4.60656 | 9.71216 | 0.00234 | 0.05 | 1 | 16.6461 | 46.62529 |
| Modified PHB - Neat PHB | -0.40944 | 5.15029 | 0.11243 | 0.99652 | 0.05 | 0 | -17.1683 | 16.34943 |

*Sig 1 means the difference between the means is significant at the significant level of 0.05*
*Sig 0 means the difference between the means is not significant at the significant level of 0.05*

**Table S6.** Statistical analysis of alamarBlue assay on day 5.

| Tukey Test | Mean Diff | SEM | q Value | Prob | Alpha | Sig | LCL | UCL |
|---|---|---|---|---|---|---|---|---|
| Neat PHB - Control | 47.0581 | 10.83881 | 6.13999 | 0.0169 | 0.05 | 1 | 11.78896 | 82.32724 |
| Modified PHB - Control | 113.9248 | 9.69452 | 16.61907 | 1.85E-04 | 0.05 | 1 | 82.37909 | 145.4705 |
| Modified PHB - Neat PHB | 66.86667 | 10.83881 | 8.72455 | 0.00379 | 0.05 | 1 | 31.59753 | 102.1358 |

*Sig 1 means the difference between the means is significant at the significant level of 0.05*
*Sig 0 means the difference between the means is not significant at the significant level of 0.05*
